# Knowledge, attitude, and practice regarding antibiotic use and resistance among medical students in Colombia: a cross-sectional descriptive study

**DOI:** 10.1186/s12889-020-09971-0

**Published:** 2020-12-04

**Authors:** Luis Felipe Higuita-Gutiérrez, Gustavo Eduardo Roncancio Villamil, Judy Natalia Jiménez Quiceno

**Affiliations:** 1 Facultad de Medicina, Universidad Cooperativa de Colombia, Escuela de Microbiología, Universidad de Antioquia, Medellín, Colombia; 2 Facultad de Medicina, Universidad Pontificia Bolivariana, Clinica Cardio VID, Medellín, Colombia; 3 Grupo de Investigación en Microbiología Básica y Aplicada (MICROBA), Escuela de Microbiología, Universidad de Antioquia, Medellín, Colombia

**Keywords:** Medical students, Antibiotics, Antibiotic resistance, Colombia

## Abstract

**Background:**

This study was designed to describe the knowledge, attitude, and practice regarding antibiotic use and resistance among medical students in Medellín, Colombia.

**Methods:**

A cross-sectional study was conducted among medical students from three universities from September to December 2018. The sample size was calculated, the classrooms were visited, and those students who were willing to participate were invited to do so. The data collection instrument was constructed in three stages: i) the literature was systematically reviewed, ii) the items from the studies identified were extracted, and iii) item reduction was performed with exploratory factor analysis. Data were analyzed by calculating absolute and relative frequencies and means for quantitative variables. The indexes of knowledge, attitude, and practice were transformed to a scale from 0 (worst possible score) to 100. Comparisons were performed using the Mann-Whitney U test, Kruskall-Wallis H test, and linear regressions.

**Results:**

Five hundred and thirty-two medical students were included with a response rate of 96%. Of the total participants, 49.1% reported having used antibiotics within the past year. Regarding knowledge, only 18.2% had heard of the term “antimicrobial stewardship” and 69.3% were aware that empiric antibiotic therapy contributes to antibiotic resistance. Regarding attitude, 11.6% considered that antibiotics should be discontinued as soon as symptoms disappear and 24.6% stated that it is better to prescribe broad-spectrum antibiotics to ensure that the patient is cured. Regarding practice, 28.5% recognized that resistance is a multifactorial problem, but they do not act on it because they consider that individual actions would have little impact. The adjusted linear regression showed that the variables associated with knowledge, attitude, and practice were socioeconomic status, training cycle, university, previous experience of research or education, the general perception of the training received, and antibiotic consumption.

**Conclusion:**

Knowledge, attitude, and practice differ widely depending on the university, training cycle, and socioeconomic status, and a significant proportion of students consider that the standard of training received at the university on antibiotics and bacterial resistance is poor or mediocre. These findings show that there is a need to strengthen the medical students’ curriculum on antibiotics, mechanisms of antibiotic resistance, and the prudent use of antibiotics as an important strategy to combat problem-resistant public health, primarily in endemic countries.

**Supplementary Information:**

The online version contains supplementary material available at 10.1186/s12889-020-09971-0.

## Background

Antibiotic resistance is a global public health concern because it adversely affects treatment results, prolongs morbidity, increases hospital stay, elevates the risk of mortality, and escalates medical costs [1]. The complexity of the situation increases when we consider that antibiotic resistance can be caused by various factors, such as medication errors; lack of or low adherence rates to therapeutic protocols based on local sensitivities; self-medication [2–4]; weak infection control systems; the use of antibiotic growth promoters in the agricultural livestock industry; wastewater pollution [5]; and limited incentives for new drug surveillance, research, and innovation [6].

In this context, health professionals play a key role in the fight against antibiotic resistance because they play a part in prescribing antibiotics during routine clinical practice as well as in promoting health education, particularly by encouraging patients to adhere to therapies and avoid self-medication [7]. Unfortunately, the inappropriate prescription of antibiotics by health professionals has proven to be a constant problem [8]. Some studies have shown that even within hospitals, the mere indication for antibiotic treatment (administration or non-administration); the choice of agent (which antibiotic to prescribe); or the dosage (posology, administration interval, and therapy duration) may be incorrect in 30–50% of cases and that in the case of intensive care units, the prescribed antibiotics are unnecessary, inappropriate, or suboptimal in 30–60% of cases. For instance, in China, more than 60% of prescriptions are inappropriate [4], and in the United States, antimicrobials are prescribed in more than 10% of outpatient consultations, one in four of which is prescribed for clinical conditions without any indications [9]. In Europe, up to 45% of doctors prescribe antibiotics for the treatment of viral infections, even when these drugs have been demonstrated to have no effect on such diseases [3].

Some authors have attributed the shortcomings in health professionals’ knowledge and practice to poor training during their undergraduate studies [7]. In this regard, a study in China reported that only 25% of medical students received specific training on the use of antimicrobials and antibiotic resistance [10]. In Italy, a study showed that approximately 20% of respondents regarded antibiotics as appropriate for viral infections, whereas 15% discontinued the prescribed treatment once the symptoms had disappeared [11]. Therefore, doctors undergoing training are a particularly relevant population when it comes to emphasizing the importance of rational antibiotic prescription and the fight against antibiotic resistance [12].

A previous study explored the knowledge, attitudes, and practices among 317 medical students in the United States, with the results indicating that 90% of the students desire more education on the appropriate use of antimicrobials, and only 33% perceived their preparedness to be adequate in some fundamental principles of antimicrobial use [13]. A survey in 13 European countries found large differences in teaching important principles on the prudent use of antibiotics [14], and another study in Congo, Africa found that knowledge regarding antibiotics was low [15]; however, in our country, despite antibiotic resistance being a substantial problem [16–19], no studies have described the reality of the training that primary antimicrobial prescribers have undergone.

Medellín is the second-largest city in Colombia, with more than 15 tertiary hospitals. In many of these hospitals and even within the community, the endemic circulation of antibiotic-resistant Gram-negative bacilli has been established by the use of both extended-spectrum β-lactamase and carbapenemase [17, 18]. High consumption of carbapenem has been shown to be associated with this problem (in 2011, meropenem consumption reached up to 30.1 DDD/100 beds/day) [19]. To tackle this issue, it has become necessary to intervene in various sectors. It is evident that education on the correct prescription, control, and prevention of infections are the bases for rectifying this problem. Some national programs have been designed to improve the administration of antibiotics [14]. These interventions are often aimed at causing behavioral changes; however, it is recognized that their impact is affected by pre-existing beliefs and motivations in each population. In this sense, for interventions to be successful and for changes to be sustained over time, the knowledge, attitudes, and practices of each stakeholder must be modified, which makes it necessary to know the baseline before implementing a program [7]. Currently, the city has six medical schools, but its handling of the antimicrobial resistance problem in terms of prescribers undergoing training remains unknown. Therefore, this study was designed with the objective of describing the knowledge, attitude, and practice regarding antibiotic use and resistance among medical students in Medellín.

## Methods

### Study design

This was a cross-sectional descriptive study.

### Subjects

A total of 532 medical students from two private universities and one public university from all semesters who voluntarily agreed to participate were included in the study. The students were invited to participate from September to December 2018. The sample size was calculated based on a reference population of 3324 medical students in the three universities, an expected standard deviation of 12 points on the scale, confidence level of 95%, sampling error of 1%, and sampling correction of 10%. The classrooms were visited and all students who were willing to participate were invited to do so. In total, 554 students were approached and 96% answered the survey. The students were selected so that all training cycles were represented. Training cycles were grouped into basic, clinical, or professional and residency stage. The basic cycle included students from semesters I to V, during which training on scientific knowledge and the morphological and functional foundations for medical actions is provided. The clinical or professional cycle included students from semesters VI to X, during which the doctor-patient relationship begins, and skills and abilities in medical practice are developed, with small groups of students attending hospital institutions under the guidance of a professor. These training cycles are then supplemented with various academic sessions (master classes, tutoring classes, or research). The remaining semesters correspond to residency, which involves in-service training with greater responsibility for patient care [20].

### Data collection

The data collection instrument was constructed in three stages. In the first stage, a systematic review of literature was conducted using the terms “antibiotic” or “antimicrobial,” each in conjunction (Boolean “&”) with the terms “survey or questionnaire” and “knowledge or belief.” The PubMed, Science Direct, EMBASE, Ovid, Scopus, Lilacs, Scielo, and Google Scholar databases were selected as data sources. The search was carried out without time restrictions. Some of the algorithms used were as follows: (((antibiotic or antimicrobial)) AND (survey or questionnaire)) AND (knowledge or belief) and (TITLE-ABS-KEY (antibiotic OR antimicrobial) AND TITLE-ABS-KEY (survey OR questionnaire) AND TITLE-ABS-KEY (knowledge OR belief)). Seven articles assessing these aspects in student health areas [7, 21–26] were identified using this strategy.

In the second stage, the items from the studies identified in the previous stage were extracted. Overall, 146 items were identified, and redundant items were eliminated. Subsequently, face validity was assessed by a multidisciplinary group of judges with training in education, antibiotic resistance, and infectiology, and the document version to be implemented was constructed. In the third stage, item elimination was performed using three statistical criteria: (1) in an exploratory factor analysis, the items with coefficients λ <  0.2 were eliminated, (2) items with the same correlation degree with the factors extracted were eliminated, and (3) Pearson’s correlations were performed with the items grouped within a component of the exploratory factor analysis to identify statistically redundant questions (correlations ≥0.8), and the item with the lowest λ coefficient was eliminated. Thus, the final instrument contained four sections. The first section accounts for sociodemographic features, antibiotic consumption, and perceptions of education received on the topic. The definition of socioeconomic status was selected in accordance with the National Administrative Department of Statistics (DANE for its acronym in Spanish). The second section contains a knowledge index, the third an attitude index, and the fourth an index of general practice on antibiotics and antibiotic resistance; each index was assessed with 10 items on a 4-point Likert scale. The questionnaire was developed in Spanish and self-administered to the students.

### Data analysis

Data were analyzed by calculating the absolute and relative frequencies and means for quantitative variables (position, dispersion, and central tendency). The indexes of knowledge, attitude, and practice were transformed to a scale ranging from 0 (worst possible score) to 100 (best possible score) as follows: Total score = [(score obtained−lowest possible score)/(maximum possible score − minimum possible score)] × 100. A higher score indicates a better performance in that domain. The total score was presented as median (interquartile range). The indexes of knowledge, attitude, and practice were compared based on sociodemographic features (sex, socioeconomic status, parents’ education) antibiotic consumption (antibiotic consumption in the past year, past six months, and past month), and education received (university, training cycle, perception of the training received so far on the subject) using the Mann-Whitney U test and Kruskall-Wallis H test after verification of non-compliance with the assumption of normality assessed using the Kolmogorov-Smirnov test with Lilliefors correction. Finally, linear regression was performed for each index to determine whether the associations found in the bivariate analysis were misleading to the extent that the variables included in the model were those with a *p*-value of < 0.05 in the bivariate analysis. All analyses were performed using SPSS version 25.0, and *p* <  0.05 was considered statistically significant.

## Results

### Sociodemographic features, antibiotic consumption, and perceptions of education received on the topic (Table 1)

A total of 532 medical students (61.1% females; 64.7% with middle socioeconomic status) were included in the study (Table 1). The response rate was 96%. The training cycle of the students was 40.8% basic, 28.3% professional, and 30.9% residency; 68.4% were affiliated to private universities. Most of the students intended to pursue postgraduate studies, primarily in surgical fields (19.6%), internal medicine (13.8%), and pediatrics (13.3%). The parents’ educational level was predominantly undergraduate and/or postgraduate education (51.7% fathers and 50.8% mothers).

**Table 1:** Sociodemographic features of the students and use of antibiotics

		Total	
		n	%
Sex	Female	325	61.1
	Male	207	38.9
Socioeconomic level according to DANE	Low	47	9.4
	Middle	322	64.7
	High	129	25.9
Father’s education level	Primary/Secondary	190	39.7
	Technical/Technological	41	8.6
	Undergraduate/Postgraduate	247	51.7
Mother’s education level	Primary/Secondary	158	32.5
	Technical/Technological	81	16.7
	Undergraduate/Postgraduate	247	50.8
He/she is planning to pursue postgraduate studies		510	96.8
He/she has experience in research or training on antibiotics and/or antibiotic resistance		291	54.7
Stage at which he/she believes that the faculty should devote more time to the subject	First year (Semester I and II)	70	13.4
	Second year (Semester III and IV)	120	23.0
	Third year (Semester V and VI)	147	28.2
	Fourth year (Semester VII and VIII)	66	12.7
	Fifth year (Semester IX and X)	59	11.3
	Sixth year (Semester XI and XII)	59	11.3
He/she has received antibiotics in the past year		261	49.1
He/she has received antibiotics in the past 6 months		171	32.3
He/she has received antibiotics in the past month		65	12.2

When asked about past antibiotic usage, 49.1% of the students reported having used them within the past year, 32.3% within the past semester, and 12.2% within the past month (Table 1). The main reasons for receiving antimicrobials included having a cough/cold/flu (20.1%), skin and soft tissue infections (16.6%), urinary tract infection (12.8%), diarrhea or other gastrointestinal problems (8.6%), and fever (3.8%). Regarding the perception of the quality of education received at the university on the topic, only 15.6% rated the training as excellent, 29.9% rated it as mediocre, or poor, 48.4% rated it as good, and 6.1% not received.

### Knowledge

**Fig. 1:**
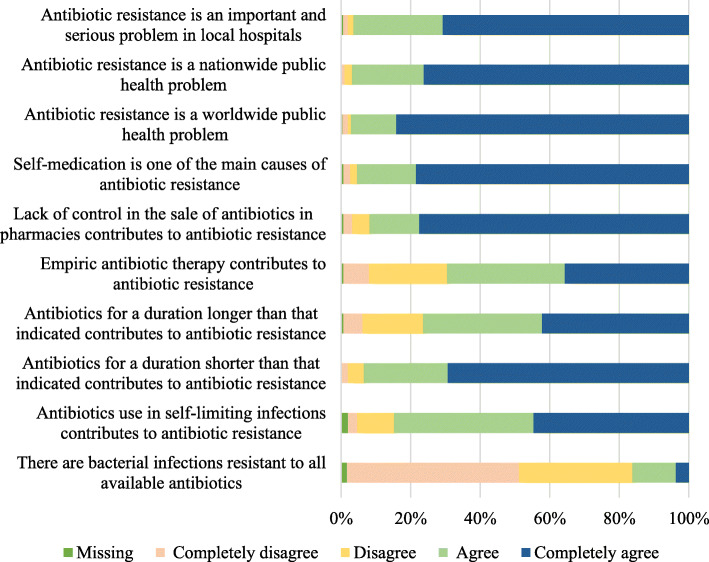
Relative frequencies of knowledge about antibiotics and antibiotic resistance

In the knowledge index, 16.2% (*n* = 87) of students were unaware that there are bacterial infections resistant to all available antibiotics (Fig. 1), only 18.2% (*n* = 97) had heard of the term “antimicrobial stewardship,” and 69.3% (*n* = 369) were aware that empiric antibiotic therapy contributes to antibiotic resistance. Notably, 97.9% (*n* = 521) of the students considered antibiotic resistance as a public health concern both internationally and locally, 95.3% (*n* = 507) recognized that self-medication is one of the main causes of antibiotic resistance, and 91.9% (*n* = 489) stated that the lack of control on the sale of antibiotics in pharmacies contributes to antibiotic resistance. When comparing the responses across universities, University C showed a better performance across all items assessed, except for the recognition of the contribution of empiric therapy to antibiotic resistance.

When transforming the responses to a scale from 0 to 100, the median knowledge was found to be 86.7 (73.3–93.3) points. This score was significantly (*p* <  0.001) lower among students with a low socioeconomic status [80.0 (70.0–83.3)] than among those with a middle [86.7 (76.7–93.3)] or high socioeconomic status [90.0 (83.3–93.3)] (Table 2); the score was lower (*p* <  0.001) among students in the basic cycle [83.3 (70.0–90.0)] than among those in the professional cycle [88.3 (81.7–93.3) or residency [86.7 (80.0–93.3)].

**Table 2:** Comparison of knowledge, attitude, and practice regarding antibiotic therapy based on sociodemographic features and antibiotic consumption

	Knowledge	Attitude	Practice
	Mean (IQR)	Mean (IQR)	Mean (IQR)
**Sex**			
Female	86.7 (76.7–93.3)	86.7 (76.7–93.3)	86.7 (73.3–93.3)
Male	86.7 (76.7–93.3)	86.7 (76.7–93.3)	86.7 (76.7–96.7)
**Socioeconomic level according to DANE**			
Low	80.0 (70.0–83.3)^**^	83.3 (70.0–90.0)^**^	83.3 (73.3–90.0)^**^
Middle	86.7 (76.7–93.3)^**^	86.7 (76.7–95.0)^**^	86.7 (73.3–93.3)^**^
High	90.0 (83.3–93.3)^**^	90.0 (80.0–96.7)^**^	90.0 (80.0–96.7)^**^
**Training cycle**			
Basic	83.3 (70.0–90.0)^**^	80.0 (66.7–90.0)^**^	73.3 (60.0–86.7)^**^
Professional	88.3 (81.7–93.3)^**^	90.0 (83.3–96.7)^**^	93.3 (83.3–96.7)^**^
Residency	86.7 (80.0–93.3)^**^	90.0 (83.3–96.7)^**^	90.0 (83.3–96.7)^**^
**Father’s education level**			
Primary or Secondary school	86.7 (76.7–93.3)	86.7 (76.7–93.3)	86.7 (73.3–93.3)
Technical or Technological	86.7 (80.0–93.3)	90.0 (83.3–93.3)	86.7 (80.0–96.7)
Undergraduate/Postgraduate	86.7 (76.7–93.3)	86.7 (76.7–93.3)	86.7 (76.7–93.3)
**Mother’s education level**			
Primary or Secondary school	83.3 (73.3–93.3)	83.3 (76.7–93.3)^**^	86.7 (73.3–93.3)
Technical or Technological	90.0 (80.0–93.3)	90.0 (80.0–96.7)^**^	86.7 (80.0–96.7)
Undergraduate or Postgraduate	86.7 (80.0–93.3)	86.7 (80.0–93.3)^**^	86.7 (76.7–93.3)
**He/she has received antibiotics in the past year**			
No	86.7 (80.0–93.3)	88.3 (80.0–96.7)	86.7 (76.7–96.7)^**^
Yes	86.7 (76.7–93.3)	86.7 (73.3–93.3)	83.3 (70.0–93.3)^**^
**He/she has received antibiotics in the past 6 months**			
No	86.7 (80.0–93.3)	90.0 (80.0–93.3)	86.7 (76.7–95.0)^**^
Yes	85.0 (76.7–93.3)	86.7 (73.3–93.3)	83.3 (70.0–93.3)^**^
**He/she has received antibiotics in the past month**			
No	86.7 (80.0–93.3)	86.7 (76.7–96.7)^**^	86.7 (76.7–93.3)^**^
Yes	83.3 (73.3–93.3)	83.3 (65.0–90.0)^**^	80.0 (56.7–90.0)^**^

^**^ *p*-value < 0.01

In addition, this score was significantly (*p* <  0.001) lower among students without any experience in research or education [83.3 (73.3–90.0)] (Table 3), those from University A (*p* = 0.001) [83.3 (73.3–93.3)], those with a poor perception of the education received on the topic (p <  0.001) [83.3 (76.7–93.3)], and those who did not receive education on this topic (p <  0.001) [76.7 (60.0–83.3)].

**Table 3:** Comparison of knowledge, attitude, and practice regarding antibiotic therapy based on the perceptions of training received on the topic

	Knowledge	Attitude	Practice
	Mean (IQR)	Mean (IQR)	Mean (IQR)
**He/she has experience in research or training on the subject**			
No	83.3 (73.3–90.0)^**^	83.3 (70.0–90.0)^**^	80.0 (63.3–90.0)^**^
Yes	86.7 (80.0–93.3)^**^	90.0 (83.3–96.7)^**^	90.0 (80.0–96.7)^**^
**He/she is planning to pursue postgraduate studies**			
No	75.0 (65.0–93.3)	76.7 (63.3–90.0)^**^	73.3 (60.0–86.7)^**^
Yes	86.7 (80.0–93.3)	86.7 (76.7–93.3)^**^	86.7 (73.3–93.3)^**^
**University**			
University A	83.3 (73.3–93.3)^**^	80.0 (66.7–90.0)^**^	76.7 (60.0–86.7)^**^
University B	86.7 (80.0–93.3)^**^	90.0 (80.0–93.3)^**^	90.0 (80.0–96.7)^**^
University C	86.7 (83.3–93.3)^**^	90.0 (83.3–96.7)^**^	90.0 (83.3–96.7)^**^
**University provides sufficient knowledge to determine when to initiate antibiotic therapy**			
No	83.3 (73.3–93.3)	83.3 (63.3–93.3)^**^	73.3 (60.0–83.3)^**^
Yes	86.7 (80.0–93.3)	90.0 (80.0–96.7)^**^	86.7 (76.7–96.7)^**^
**University provides sufficient knowledge to select the best antibiotic for each infection**			
No	83.3 (76.7–93.3)	90.0 (76.7–93.3)	83.3 (70.0–93.3)^**^
Yes	86.7 (80.0–93.3)	86.7 (80.0–93.3)	86.7 (76.7–96.7)^**^
**University provides sufficient knowledge to understand the basic resistance mechanisms**			
No	86.7 (76.7–90.0)	90.0 (73.3–96.7)	80.0 (70.0–93.3)^**^
Yes	86.7 (76.7–93.3)	86.7 (80.0–93.3)	86.7 (76.7–96.7)^**^
**University provides sufficient knowledge to interpret antibiograms**			
No	83.3 (76.7–93.3)	90.0 (80.0–93.3)	86.7 (73.3–96.7)
Yes	86.7 (76.7–93.3)	86.7 (76.7–96.7)	86.7 (76.7–93.3)
**University provides sufficient knowledge to find sources of information on infections**			
No	86.7 (76.7–93.3)	90.0 (76.7–93.3)	83.3 (70.0–90.0)
Yes	86.7 (80.0–93.3)	86.7 (76.7–96.7)	86.7 (76.7–96.7)
**University provides sufficient knowledge to change from intravenous antibiotics to oral antibiotics**			
No	86.7 (76.7–93.3)	90.0 (80.0–93.3)	86.7 (76.7–93.3)^**^
Yes	86.7 (76.7–93.3)	86.7 (76.7–96.7)	86.7 (73.3–93.3)^**^
**General perception of the training received so far on the subject**			
He/she has not received training	76.7 (60.0–83.3)^**^	70.0 (63.3–83.3)^**^	63.3 (50.0–73.3)^**^
Mediocre /Poor	83.3 (76.7–93.3)^**^	90.0 (76.7–93.3)^**^	83.3 (71.7–93.3)^**^
Good	86.7 (80.0–93.3)^**^	86.7 (80.0–93.3)^**^	86.7 (80.0–93.3)^**^
Excellent	90.0 (80.0–96.7)^**^	90.0 (80.0–96.7)^**^	90.0 (80.0–00.0)^**^

^**^ *p*-value < 0.01

In the linear regression model for the knowledge index (Table 4), the scores were explained in 10.6% by the socioeconomic status, training cycle, previous experience in research or education on the topic, and the general perception of the education received on the topic.

**Table 4:** Linear regression models for each index

Index	Model variables	***p*** value	Regression coefficient	Coefficient of determination
**Knowledge**	Socioeconomic level	0.002	3.223	10.6%
	Training cycle	< 0.001	2.538	
	Previous experience	0.047	2.402	
	Perception of the training	0.004	2.112	
**Attitude**	Training cycle	< 0.001	4.605	19.1%
	Antibiotic use in the past month	0.003	−5.609	
	Previous experience	0.001	4.522	
	University	0.039	2.137	
**Practice**	Training cycle	< 0.001	5.217	29.4%
	Antibiotic use in the past month	< 0.001	−6.924	
	Previous experience	0.001	4.693	
	University	0.023	2.323	
	General perception of the education	< 0.001	3.510	

### Attitude

**Fig. 2:**
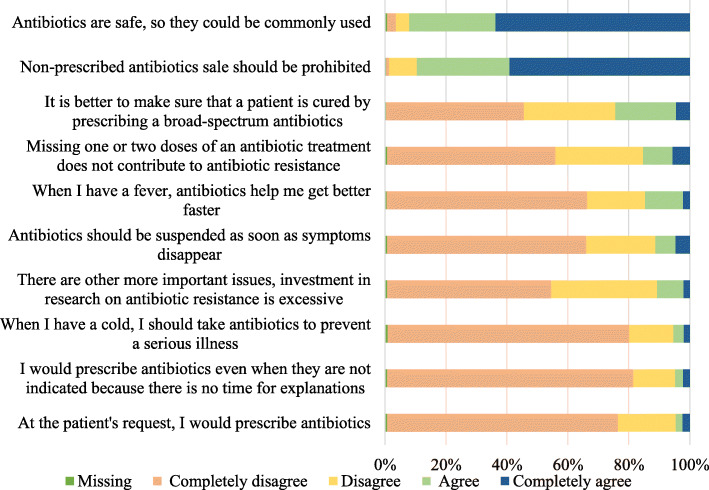
Relative frequencies of attitude on antibiotics and antibiotic resistance

Among the most important results on attitude (Fig. 2), 11.6% (*n* = 62) of the students considered that antibiotics should be discontinued as soon as symptoms disappear, and 24.6% (*n* = 131) stated that it is better to prescribe broad-spectrum antibiotics to ensure that the patient is cured of the infection. Additionally, 14.8% (*n* = 79) considered that antibiotics help resolve a fever faster, 92.4% (*n* = 492) considered antibiotics as safe drugs that can be used commonly, and only 59.4% (*n* = 316) considered it important to wait for the culture results before initiating antibiotic therapy. Of note, 90% (*n* = 479) considered that the sale of non-prescribed antibiotics should be prohibited.

The scores in this index were lower among students with a low socioeconomic status (*p* = 0.005) [83.3 (70.0–90.0)] (Table 2), students in the basic cycle (*p* <  0.001) [80.0 (66.7–90.0)], those whose mother’s educational level was low (*p* = 0.013) [83.3 (76.7–93.3)], and those who had received antibiotics in the past month (*p* = 0.003) [83.3 (65.0–90.0)]. The scores were also significantly lower among students who were not planning to pursue postgraduate studies (*p* = 0.029) [76.7 (63.3–90.0)] (Table 3) and among those who perceived that the university does not provide sufficient information regarding when to initiate antibiotic therapy (*p* = 0.010) [83.3 (63.3–93.3)].

In the linear regression model for the attitude index (Table 4), scores were explained in 19.1% by the training cycle, antibiotic consumption in the past month, previous experience or education on the topic, and university.

### Practice

**Fig. 3:**
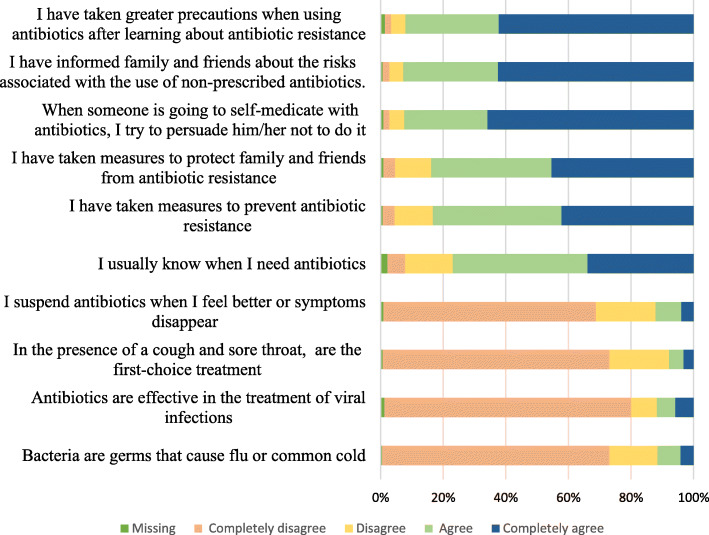
Relative frequencies of practice regarding antibiotics and antibiotic resistance

Regarding practice, 11.8% (*n* = 63) of the students stated that antibiotics are effective for treating viral infections (Fig. 3), 11.6% (*n* = 62) stated that antibiotics are used to treat flu or the common cold, 12.2% (*n* = 65) stated that antibiotics should be discontinued when symptoms disappear, 8% (*n* = 43) stated that antibiotics are the first-choice treatment in the presence of cough and sore throat, and 28.5% (*n* = 152) recognized that antibiotic resistance is a multifactorial problem but do not act on it because individual actions would have little impact. This index highlighted that students had taken greater precautions regarding antibiotic use [93.3% (*n* = 496)] after learning about antibiotic resistance, they had informed their family and friends about the risks associated with non-prescribed antibiotics [93.4% (*n* = 497)], and, when confronted with self-medication, they attempted to persuade people not to do so [93.2% (n = 496)].

When comparing the scores (Table 2), practice scores were noted to be worse among students with a low socioeconomic status (*p* < 0.001) [83.3 (73.3–90.0)]; those in the basic cycle (*p* < 0.001) [73.3 (60.0–86.7)]; and those who had received antibiotics within the past year (*p* = 0.012) [83.3 (70.0–93.3)], past six months (*p* = 0.027) [83.3 (70.0–93.3)], and past month (*p* = 0.001) [80.0 (56.7–90.0)]. Similarly, the scores were lower among those with no experience in research or education on the topic (*p* < 0.001) [80.0 (63.3–90.0)] (Table 3); those who were not planning to pursue postgraduate studies (*p* = 0.014) [73.3 (60.0–86.7)]; those from University A (*p* < 0.001) [76.7 (60.0–86.7)]; those who considered that the university does not provide sufficient knowledge regarding when to initiate antibiotic therapy (*p* < 0.001) [73.3 (60.0–83.3)], select the ideal antibiotic for each specific infection (*p* = 0.015) [83.3 (70.0–93.3)], and understand the basic mechanisms of resistance (*p* = 0.007) [83.3 (70.0–93.3)]; those who did not receive training on the topic (*p* < 0.001) [63.3 (50.0–73.3)]; and those who perceived that the quality of education was mediocre or poor (*p* < 0.001) [83.3 (71.7–93.3)].

In the linear regression model for the practice index (Table 4), scores were explained in 29.4% by the training cycle, antibiotic consumption in the past month, previous experience or education on the topic, university, and general perception of the training received.

## Discussion

This study showed that knowledge, attitude, and practice regarding antibiotics and antibiotic resistance are generally good to the extent that they can be represented by scores of > 80 points on a scale from 0 to 100; however, they markedly vary depending on the university, training cycle, and socioeconomic status.

In this study, attitude and practice differed among universities, and this effect continued even after multivariate adjustment. A similar study that assessed 579 pharmaceutical science students found that knowledge and attitude also vary depending on the university [24]. This result suggests that some universities are preparing their students more consistently regarding the particular topic of antibiotics and antibiotic resistance, which may provide an opportunity to seek consensus among universities regarding the expertise that medical students should develop [24]. In this context, using the Delphi technique, a group of researchers from the United Kingdom developed a national consensus on the competencies in the use of antibiotics that health professionals should develop. These competencies were grouped into eight domains that accounted for the prevention and control of infections, antibiotics and antibiotic resistance, infection diagnosis and antibiotic use, antibiotic prescription practices, person-centered care, and the promotion of collaborative practice among various professionals [27]. This scenario constitutes the basis for working with a national consensus that adapts the proposed aspects in the United Kingdom to the local epidemiological context.

Another factor associated with knowledge, attitude, and practice was the training cycle; students in the basic cycle obtained lower scores in all the indexes evaluated. This is consistent with the findings of another study that assessed Chinese medical students, which reported that students’ knowledge improved as they progressed in their studies, with the highest scores obtained in the third and fourth years [28]. The explanation for this finding could be that pharmacology, internal medicine, and epidemiology are the subjects that are not offered to students during their first year of study and are fundamental within the curriculum in the consolidation of knowledge on the use of antibiotics and the problems of antibiotic resistance [28]. However, this is not always the case. A study conducted among doctors at the Johns Hopkins University Hospital in Baltimore, USA, showed that doctors had suboptimal knowledge about antimicrobials and their knowledge did not improve during the training course [29]. This shows that training itself does not improve knowledge; thus, a rigorous evaluation of the content is necessary, and didactic and pedagogical strategies need to be implemented so that it is not merely a marginal element within the curriculum but rather something that must continually be referenced from the initial semesters until the final semester.

Consistent with the above results, those who perceived that the training received was excellent obtained significantly higher scores in all the indexes evaluated. This is a relevant finding because medical students’ perceptions of the quality of the training received coincide with their level of knowledge, which constitutes a good indirect indicator of the education received. In the present study, only 15.6% of the students rated the education received at the university as excellent and 29.9% as mediocre or poor. A recent review of educational programs promoting the prudent use of antibiotics revealed that most educational efforts have been directed at medical professionals and have proven to be effective in reducing antibiotic prescription; however, such programs are less common among health professionals [30]. Thus, it is crucial to develop appropriate curricula to teach microbial virulence, antibiotic resistance mechanisms, and prudent antibiotic use to university medical students as well as students of other disciplines (pharmacy, dentistry, nursing, veterinary medicine, and microbiology) [30].

The socioeconomic status was associated with knowledge, and this association was maintained even after multivariate adjustment. This finding can be attributed to the fact that a population with a low socioeconomic status has greater barriers in accessing the health system, which discourages consultations and promotes self-medication. Self-medication generally occurs as a result of the recommendations of local friends and pharmacists, who may have misconceptions and inappropriate practices about antibiotic use and resistance [31]. In this context, popular knowledge of antibiotics is deeply rooted in culture and becomes difficult to modify with subsequent interventions. Additionally, previous studies found an association between socioeconomic status and antibiotic use and resistance [32, 33]. This indicates that socioeconomic status is indeed a determining factor for both individual behavior and health conditions of the population.

Antibiotic consumption among medical students ranged from 48.9% in the past year to 12.1% in the past month and was most frequent for coughs/colds/flu and to a lesser extent for fever. A study that evaluated 1042 medical students from two universities in Saudi Arabia found that almost 97.2% of the students had used antibiotics in the previous year, 49% reported self-medication, and 61.8 and 18.1% thought that antibiotics could be used for the symptoms of the urinary tract and viral infections, respectively [34]. Several researchers agree that it is necessary to provide more information about antibiotics and the possible adverse effects of their indiscriminate use because such information helps reduce the frequency of use and encourages the proper use of these drugs. However, it has also been documented that knowledge does not always correlate with behavior [21]. Apparently, the latter occurs within the context of this study, while the practice of medical students coincides with what has been reported in the general population. In a study of 1141 adults, 67.1% believed that antibiotics can be used to treat common colds and coughs, 28.1% used antibiotics as an analgesic, 55.6% used them as prophylaxis against infections, and 9.7% believed that they are equivalent to antipyretics and thus useful to resolve a fever [35]. This proves that regarding knowledge, there is a notion that antibiotics are *miracle drugs*—a belief that has been internalized for generations and would account for the healing properties attributed to them in treating infectious and non-infectious diseases as well as their careless and confident broad usage, both among medical students and the general population [36]. Consistent with this, this study found that 91.9% of students consider that the lack of control over the sale of antibiotics in pharmacies contributes to antibiotic resistance. In this regard, it is important to keep in mind that even though there are regulations in the country that restrict the sale of antibiotics to the presentation of a medical prescription, a study carried out in Bogotá, Colombia’s capital, showed that of the total of pharmacies studied, 80.3% sold antibiotics without a prescription. In 20.1% of these cases, the dispenser asked about the patient’s age and symptoms to offer him other medications or change the antibiotic. Additionally, some drug dispensers made inappropriate recommendations, which reflects that this topic is of particular importance in the fight against bacterial resistance [37].

Another interesting research finding is that 97% of the students believed that antibiotic resistance is a public health issue, both internationally and locally. This finding could be attributed to two factors. In addition to the recognition of the problem within the university context and its incorporation into the micro curriculum, antibiotic resistance is being widely discussed and publicized in the press, television, and other media; therefore, raising public awareness has become easy. For young individuals, in particular, the media and the Internet have become important sources of information and the means of acquiring knowledge and awareness of various public issues [7].

Our study has several limitations. As the measurements were transversal, the associations found do not indicate causality. The survey was a self-administered questionnaire; thus, the use of antibiotics was basically self-reported, and the frequency of antibiotic use may be overestimated or underestimated due to recall bias. Finally, the results are only representative of medical students from three universities in the city.

## Conclusions

Knowledge, attitude, and practice regarding antibiotics and antibiotic resistance differ widely depending on the university, training cycle, and socioeconomic status. Antibiotics are commonly used by medical students in the city, and a significant proportion rated the training received at the university on the topic as mediocre or poor. These findings show that there is a need to strengthen the medical students’ curriculum on antibiotics, mechanisms of antibiotic resistance, and the prudent use of antibiotics as an important strategy to combat problem-resistant public health.

## Supplementary Information

**Additional file 1: Supplementary file questionnaire**. Questionnaire “Survey on Knowledge, Attitudes, and Practices (KAP) related to antibiotics and bacterial resistance”. Questionnaire applied to students during the study.

## Data Availability

The datasets used and analysed during the current study are available from the corresponding author on reasonable request.
