# Supplementary material for: Knowledge, attitude, and practice regarding antibiotic use and resistance among medical students in Colombia: a cross-sectional descriptive study
Source: BMC Public Health. 2020 Dec 4;20:1861. doi: 10.1186/s12889-020-09971-0 (PMC7718705; doi:10.1186/s12889-020-09971-0)
Supplement: Supplementary file 1 — Additional file 1: Supplementary file questionnaire. Questionnaire “Survey on Knowledge, Attitudes, and Practices (KAP) related to antibiotics and bacterial resistance”. Questionnaire applied to students during the study. [file 12889_2020_9971_MOESM1_ESM.docx]

**Survey on Knowledge, Attitudes, and Practices (KAP) related to antibiotics and bacterial resistance.**

The data requested in this survey is confidential. Under no circumstances will your identity be requested and the information obtained here may only be used for investigative purposes. The results will be published through statistical aggregates. We ask you to answer honestly questions honestly that we will ask you below, remember that the answer is anonymous.

| **1.**Code_____ | **2.**University___________________________ |
| --- | --- |
| **3.Age**:__________(Years). | .Sexo: **4.**Semester ____________________________ |
| **5.**Sex: Female ☐ Male ☐ | **6.** What is your socioeconomic status?_______ |
| **7.** Until what year did your father study_________ | **8.** Until what year did your mother study__________ |
| **9.** Have you had any research or education experience regarding antibiotics and/or bacterial resistance?  Yes☐ No ☐ | **10.** Are you thinking of pursuing graduate studies?  Yes ☐ No ☐  What specialty are you considering?____________ |
| **11.** Have you taken antibiotics in the last year?  Yes ☐ No ☐  **12.** Have you taken antibiotics in the six months? Yes ☐ No ☐  **13.** Have you taken antibiotics in the last month?  Yes ☐ No ☐ | **14.** What was the illness for which you took antibiotics?  Cough/Cold/Flu☐ Fever☐  Other respiratory problems ☐  Wound infection ☐ Ear/eye infection ☐  Diarrhea or other gastrointestinal problems ☐  Other, Which?________________ |
| **17.** How would you rate your education so far on antibiotic use and bacterial resistance?  Excellent ☐ Good ☐ Regular ☐ Poor ☐ I have not received any education so far ☐ | |
| **18.** Do you think university sufficiently prepares to do the following after graduation?  Know when to start antibiotic therapy Yes☐ No☐  How to select the best antibiotic for each specific infection Yes☐ No☐  Understand the basic mechanisms of antibiotic resistance Yes☐ No☐  Know how to interpret antibiograms Yes☐ No☐  How to find reliable sources of information to treat infections Yes☐ No☐  How to switch from intravenous antibiotics to oral antimicrobials Yes☐ No☐ | |
| **19.** In which year do you think your faculty should spend the most time teaching antibiotic use and bacterial resistance?  First year ☐ Second year ☐ Third year ☐ Fourth year ☐ Fifth year ☐ Sixth year ☐  None of the above ☐ | |

**Knowledge**

In the following statements check **Completely disagree**, **Disagree**, **Agree,** or **Completely agree**

| **Item** | **Completely disagree** | **Disagree** | **Agree** | **Completely agree** |
| --- | --- | --- | --- | --- |
| There are bacterial infections resistant to all available antibiotics |  |  |  |  |
| Antibiotic use in self-limiting infections contributes to antibiotic resistance |  |  |  |  |
| Antibiotics for a duration shorter than that indicated contributes to antibiotic resistance |  |  |  |  |
| Antibiotics for a duration longer than that indicated contributes to antibiotic resistance |  |  |  |  |
| Empiric antibiotic therapy contributes to antibiotic resistance |  |  |  |  |
| Lack of control in the sale of antibiotics in pharmacies contributes to antibiotic resistance |  |  |  |  |
| Self-medication is one of the main causes of antibiotic resistance |  |  |  |  |
| Antibiotic resistance is a worldwide public health problem |  |  |  |  |
| Antibiotic resistance is a nationwide public health problem |  |  |  |  |
| Antibiotic resistance is an important and serious problem in local hospitals |  |  |  |  |

**Attitudes**

In the following statements check **Completely disagree**, **Disagree**, **Agree,** or **Completely agree**

| **Item** | **Completely disagree** | **Disagree** | **Agree** | **Completely agree** |
| --- | --- | --- | --- | --- |
| At the patient's request, I would prescribe antibiotics |  |  |  |  |
| I would prescribe antibiotics even when they are not indicated because there is no time for explanations |  |  |  |  |
| When I have a cold, I should take antibiotics to prevent a serious illness |  |  |  |  |
| There are other more important issues. Investment in research on antibiotic resistance is excessive |  |  |  |  |
| Antibiotics should be suspended as soon as symptoms disappear |  |  |  |  |
| When I have a fever, antibiotics help me get better faster |  |  |  |  |
| Missing one or two doses of an antibiotic treatment does not contribute to antibiotic resistance |  |  |  |  |
| It is better to make sure that a patient is cured by prescribing broad-spectrum antibiotics |  |  |  |  |
| Non-prescribed antibiotics sale should be prohibited |  |  |  |  |
| Antibiotics are safe, so they could be commonly used |  |  |  |  |

**Practices**

In the following statements check **Completely disagree**, **Disagree**, **Agree,** or **Completely agree**

| **Item** | **Completely disagree** | **Disagree** | **Agree** | **Completely agree** |
| --- | --- | --- | --- | --- |
| Bacteria are germs that cause flu or common cold |  |  |  |  |
| Antibiotics are effective in the treatment of viral infections |  |  |  |  |
| In the presence of a cough and sore throat, antibiotics are the first-choice treatment |  |  |  |  |
| I suspend antibiotics when I feel better or symptoms disappear |  |  |  |  |
| I usually know when I need antibiotics |  |  |  |  |
| I have taken measures to prevent antibiotic resistance |  |  |  |  |
| I have taken measures to protect family and friends from antibiotic resistance |  |  |  |  |
| When someone is going to self-medicate with antibiotics, I try to persuade him/her not to do it |  |  |  |  |
| I have informed family and friends about the risks associated with the use of non-prescribed antibiotics. |  |  |  |  |
| I have taken greater precautions when using antibiotics after learning about antibiotic resistance |  |  |  |  |
